# A Genome-Wide Screen Identifies Factors Involved in *S. aureus*-Induced Human Neutrophil Cell Death and Pathogenesis

**DOI:** 10.3389/fimmu.2019.00045

**Published:** 2019-01-31

**Authors:** Dingyi Yang, Yin Xin Ho, Laura M. Cowell, Iqra Jilani, Simon J. Foster, Lynne R. Prince

**Affiliations:** ^1^Department of Infection, Immunity and Cardiovascular Disease, University of Sheffield, Sheffield, United Kingdom; ^2^Florey Institute, University of Sheffield, Sheffield, United Kingdom; ^3^Department of Molecular Biology and Biotechnology, University of Sheffield, Sheffield, United Kingdom

**Keywords:** *Staphylococcus aureus*, neutrophils, cell death, methicillin resistant *S. aureus* (MRSA), zebrafish

## Abstract

*Staphylococcus aureus* is a commensal organism in approximately 30% of the human population and colonization is a significant risk factor for invasive infection. As a result of this, there is a great need to better understand how *S. aureus* overcomes human immunity. Neutrophils are essential during the innate immune response to *S. aureus*, yet this microorganism uses multiple evasion strategies to avoid killing by these immune cells, perhaps the most catastrophic of which is the rapid induction of neutrophil cell death. The aim of this study was to better understand the mechanisms underpinning *S. aureus-*induced neutrophil lysis, and how this contributes to pathogenesis in a whole organism model of infection. To do this we screened the genome-wide Nebraska Transposon Mutant Library (NTML) in the community acquired methicillin resistant *S. aureus* strain, USA300, for decreased ability to induce neutrophil cell lysis. Out of 1,920 *S. aureus* mutants, a number of known regulators of cell lysis (including the master regulators accessory gene regulator A, *agrA* and Staphylococcus exoprotein expression protein S, *saeS*) were identified in this blinded screen, providing validity to the experimental system. Three gene mutations not previously associated with cell death: *purB, lspA*, and *clpP* were found to be significantly attenuated in their ability to induce neutrophil lysis. These phenotypes were verified by genetic transductants and complemented strains. *purB* and *clpP* were subsequently found to be necessary for bacterial replication and pathogenesis in a zebrafish embryo infection model. The virulence of the *clpP* mutant was restored in a neutrophil-depleted zebrafish model, suggesting the importance of ClpP in mechanisms underpinning neutrophil immunity to *S. aureus*. In conclusion, our work identifies genetic components underpinning *S. aureus* pathogenesis, and may provide insight into how this commensal organism breaches innate immune barriers during infection.

## Introduction

*Staphylococcus aureus* has long been recognized as a highly adaptive and dangerous human pathogen, yet this microorganism colonizes the nose of ~30% of the human population without any ill effects (1). Highly virulent methicillin resistant *S. aureus* (MRSA) strains can also be carried asymptomatically by healthy individuals (2). Interventions such as hospitalization or episodes of immunosuppression can result in invasive *S. aureus* infection, which can manifest in multiple forms from superficial skin abscesses to necrotising pneumonia or life-threatening bacteraemia. Colonization is a significant risk factor for pathogenic infection (3, 4). Considering the speed and efficiency with which *S. aureus* acquires resistance to antibiotics, the shift from silent passenger to life-threatening pathogen is all the more concerning.

Neutrophils are a critical defense in controlling colonization and active infection with *S. aureus* (5, 6). Yet this microorganism uses multiple evasion strategies to avoid killing by these innate immune cells [reviewed in (7)]. Perhaps the most catastrophic of these strategies is the induction of neutrophil cell death. This not only eradicates a critical element of the early immune response, but also results in inflammation and tissue damage which intensifies disease. *S. aureus* has been shown to upregulate cell death pathway genes and promote neutrophil apoptosis as well as programmed necrosis (8–10). *S. aureus* also produces a number of cytolytic toxins including Panton-Valentine leukocidin (PVL), phenol-soluble modulins (PSMs), α-hemolysin, and the leukotoxin LukAB (11). Cytolytic toxin production is intimately linked with pathogenesis, including in community-acquired MRSA (CA-MRSA) infection (12, 13). Understanding host-pathogen interactions in the context of CA-MRSA is imperative since these strains spread rapidly between individuals and are able to cause disease in healthy people. The increased virulence of CA-MRSA has been attributed in part to its resistance to neutrophil-mediated killing, including via the induction of neutrophil lysis (14).

Previous studies have taken candidate approaches to the study of components involved in neutrophil lysis, which may have resulted in an incomplete picture of the genetics underpinning cell death. Here we have performed an unbiased genome-wide study to identify additional routes to neutrophil cell death. Using the NTML we have screened all non-essential *S. aureus* genes to identify genetic components that are involved in inducing neutrophil cell lysis. The NTML was created in the CA-MRSA strain USA300, and has been successfully applied in diverse phenotypic screens to identify genes involved in polymicrobial interactions, antimicrobial resistance, and pathogenicity (15–18). A high-throughput, flow cytometric human neutrophil cell death assay has revealed three genes (*purB, lspA*, and *clpP*) that are required for cell lysis. The role of these factors in disease has been determined using a zebrafish embryo model of infection. Our study has provided further evidence for the complex interaction between pathogen and host that determines disease outcome.

## Materials and Methods

### Bacterial Information and Culture

All *S. aureus* strains were grown in brain heart infusion (BHI) broth at 37°C with aeration at 250 rpm unless otherwise stated. USA300 *S. aureus* strain JE2 was used as a positive control. NTML strains were cultured in liquid BHI in 96-well plates. Transductant and complement strains were grown on BHI agar plates followed by overnight inoculation in 250 ml BHI broth. Where required, selection for antibiotic resistance markers was carried out using the following concentrations: ampicillin (Amp, 100 μg/ml); chloramphenicol (Cm, 30 μg/ml); erythromycin (Ery, 2.5 μg/ml); lincomycin (Lin, 12.5 μg/ml); kanamycin (Kan, 50 μg/ml); tetracycline (Tet, 5 μg/ml). The NTML was acquired from the Network on Antimicrobial Resistance in *S. aureus* (NARSA) strain repository. The NTML was constructed based on USA300 FPR3757 chromosomal genome sequence mapped transposition of *bursa aurealis* from the delivery plasmid pBursa into the non-essential protein coding sequences of the wild-type JE2 strain (15). Colony forming unit (CFU) count results were obtained using the Miles and Misra method (19). Bacterial density was quantified by spectrophotometric reading at 600 nm (OD600) using a Jenway 6100 spectrophotometer. NTML strain OD600 was measured using a Perkin VICTOR x3 2030 plate reader, with orbital shaking for 0.2 s before reading.

### Genetic Transduction and Complementation

*Staphylococcus aureus* transduction was performed with Φ11 as described previously (20). Transformation of *S. aureus* RN4220 and *E. coli* was carried out by electroporation based on previous methods (21, 22). For genetic complementation, the *lspA* operon was amplified from JE2 genomic DNA with Phusion polymerase (NEB), using primers containing appropriate restriction sites (forward, GAATTCGTACGGGCCCGGGCTTACTTAACCTCCTTCTCC; reverse, CCATGTAGGCCAAGTCAAATGAATAATTAAGTTCATATTTAATGTCAAAA). The pKASBAR-Kan^R^, a plasmid carrying an *attP* encoding site, was inserted with the PCR product to integrate the insert into the *S. aureus* genome via the *attB* site, in the presence of an integrase (23). The plasmid providing integrase, pYL112Δ19, was propagated into *S. aureus* RN4220 strain. From RN4220, the insert was transduced into *lspA* and control strains. The *clpP* mutant and Φ85 complemented *clpP*^+^ strain in 8325 background were kindly provided by Knut Ohlsen (24).

### Chemical Complementation

Chemical complementation of *purB* was conducted with adenine (20 mg/ml in 0.5 M HCl) and inosine (50 mg/ml in dH_2_O). A final concentration of 20 μg/ml was achieved in BHI agar or RPMI (+10% FCS) media accordingly.

### Neutrophil Isolation and Culture

Human neutrophils were isolated by dextran sedimentation followed by plasma-Percoll gradient centrifugation from whole blood of healthy volunteers as previously described (25, 26). Written informed consent and ethical approval from the South Sheffield Research Ethics Committee (study number STH13927) were obtained. The purity of isolated neutrophils was determined from Diff-Quik (Sigma-Aldrich, St. Louis, MO) stained cytocentrifuge preparations by light microscopy. Neutrophils were suspended at 5 × 10^6^/ml in RPMI 1640 (Thermo Fisher Scientific, Waltham, MA, United States) + 10% fetal calf serum (FCS, PromoCell, Heidelberg, Germany) and cultured in 96-well plates at 37°C, 5% CO_2_. Phagocytosis assays were performed by incubating neutrophils (in RPMI + 10% FCS) with *S. aureus* strains at a multiplicity of infection (MOI) of 10 for 1 h after which they were cytocentrifuged (Cytospin, Shandon) onto microscope slides (27). Cells were stained with Quik-Diff dyes and *S. aureus* visualized within neutrophils by oil immersion light microscopy. The phagocytic index was calculated using the following formula: (total number of engulfed bacteria/total number of neutrophils) × (number of neutrophils containing bacteria/total number of neutrophils) × 100.

### Intracellular Killing Assay

Neutrophils (4.5 × 10^5^ cells/well) were infected with *S. aureus* at an MOI of 5 at 37°C, 5% CO_2_ in RMPI + 10% FCS. The number of internalized viable *S. aureus* was determined after 30 min. Cells were centrifuged at 2000 RPM for 2 min, resuspended in 200 μl 1% saponin (in PBS), and incubated at RT for 10 min with constant vortexing to lyse neutrophils. The number of viable *S. aureus* in cell lysates was determined by the Miles and Misra method (19). In parallel wells, lysostaphin (20 μg/ml) was added for 30 min to kill extracellular *S. aureus* (28). Viable intracellular *S. aureus* was determined after a further 60 min (120 min in total) as above.

### NTML Neutrophil Cell Death Assay Screen

NTML strains were grown overnight in 96-well plates containing BHI broth following which 10 μl was sub-cultured into 190 μl BHI for 3 h at 37°C. OD_600_ measurements varied from 0.3 to 0.7. Cultures were centrifuged at 5,000 g for 10 min (RT) and pellets were resuspended in 200 μl of RPMI (+10% FCS) for use in cell death assays. Five microliters of each strain were added to 2.5 × 10^5^ neutrophils in individual wells of a 96-well plate to achieve an MOI of 10. The following conditions were also included for each experiment: media control (neutrophils in RPMI + 10% FCS) and JE2 (WT) challenge. Plates were incubated at 37°C, 5% CO_2_ for 3 h. Following this, 100 μl cold PBS containing ToPro-3 (100 nM) was added to each well and samples were immediately subjected to flow cytometry using an Attune Autosampler (ThermoFisher, Waltham, MA). To avoid lengthy plate reading times a maximum of two 96-well plates plus controls were assessed in any one experiment. Plates were acquired at a speed of 500 μl/min and stopped once 70 μl of each sample has been aspirated. Cell counts were automatically generated. Flow cytometric data was analyzed by FlowJo software (TreeStar, Ashland OR). FSC/SSC dot plot profiles of media control conditions were used to set a gate around viable neutrophils and absolute cell numbers were automatically enumerated in this gated region for all plots. Events in the viable gate were exclusively ToPro-3 negative (data not shown), verifying cell viability in this population. The genetic identity of mutant strains was not identified until after analysis was completed to avoid bias.

### Lactate Dehydrogenase Assay (LDH) Cytotoxicity Assay

Neutrophils (4.5 × 10^5^ cells/well) were incubated in media (RPMI + 10% FCS) alone or challenged with *S. aureus* strains at MOI of 5 for 3 h after which the cultures were centrifuged at 300 g for 5 min. LDH activity was quantified in 50 μl supernatant using Pierce™ LDH Cytotoxicity Assay Kit, according to the manufacturer's instructions.

### Zebrafish Models of Infection

Zebrafish embryos < 5 days postfertilization (dpf) are not protected under the Animals (Scientific Procedures) Act 1986, but all zebrafish work was carried out according to the details set out in Project License PPL 40/3574. London Wild Type (LWT) strains were maintained in E3 medium at 28°C by following standard protocols and used for all experiments (29). Embryos were dechorionated 1 day prior to bacterial injection. Zebrafish embryos at 24 hpf were anesthetized in 0.02% (w/v) tricaine for 8 min prior to bacterial injection. The stock solution of 0.4% (w/v) 3-amino benzoic acid ester tricaine (Sigma-Aldrich) was made in 20 mM tris-HCl (pH 7). *S. aureus* was microinjected into the circulation valley located ventral to the yolk sac as described previously (29). The inoculum was determined retrospectively by Miles and Misra method. Zebrafish viability was determined by visual assessment based on cessation of heartbeat and circulatory flow, and assessed at time points of 20, 26, 44, 50, 68, 74, and 92 h post-injection. Bacterial growth was assessed from homogenized embryos plated onto BHI agar.

### Morpholino Oligonucleotide Depletion of Neutrophils

Morpholino oligonucleotides to *pu.1* (sequence 5′-3′: GATATACTGATACTCCATTGGTGGT) were microinjected into the yolk sac of embryos within 30 min of fertilization (1–4 cell stage) (29, 30). Infection studies were carried out as described above. All survival studies and morpholino data were collected over 3 independent experiments, each comprising of 30 (morpholino and survival studies) or 120 (bacterial growth studies) embryos per group.

### Statistical Analysis

The Kaplan-Meier method was used to generate survival curves.

The log-rank (Mantel Cox) test was performed to compare survival curves of different strains. All statistical analysis was completed by GraphPad Prism Version 6.0 by one-way ANOVA or as otherwise stated. Significant differences were indicated as: ^*^p < 0.05, ^**^p < 0.01, ^***^p < 0.001, ns, not-significant.

## Results

### Establishing a High-Throughput *S. aureus*-Induced Neutrophil Lysis Screen

**Figure 1:**
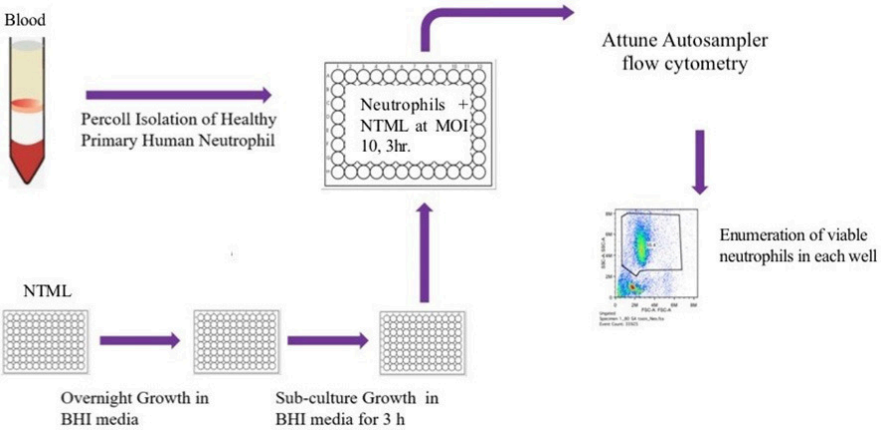
Flow diagram illustrating the NTML neutrophil cell death screen. The NTML was grown overnight in 96-well plates and sub-cultured for a further 3 h on the day of the screen. Neutrophils were isolated from human blood and infected with library strains at MOI 10 for 3 h 96-well plates were subjected to an Attune Autosampler flow cytometer for absolute numbers of viable neutrophils.

**Figure 2:**
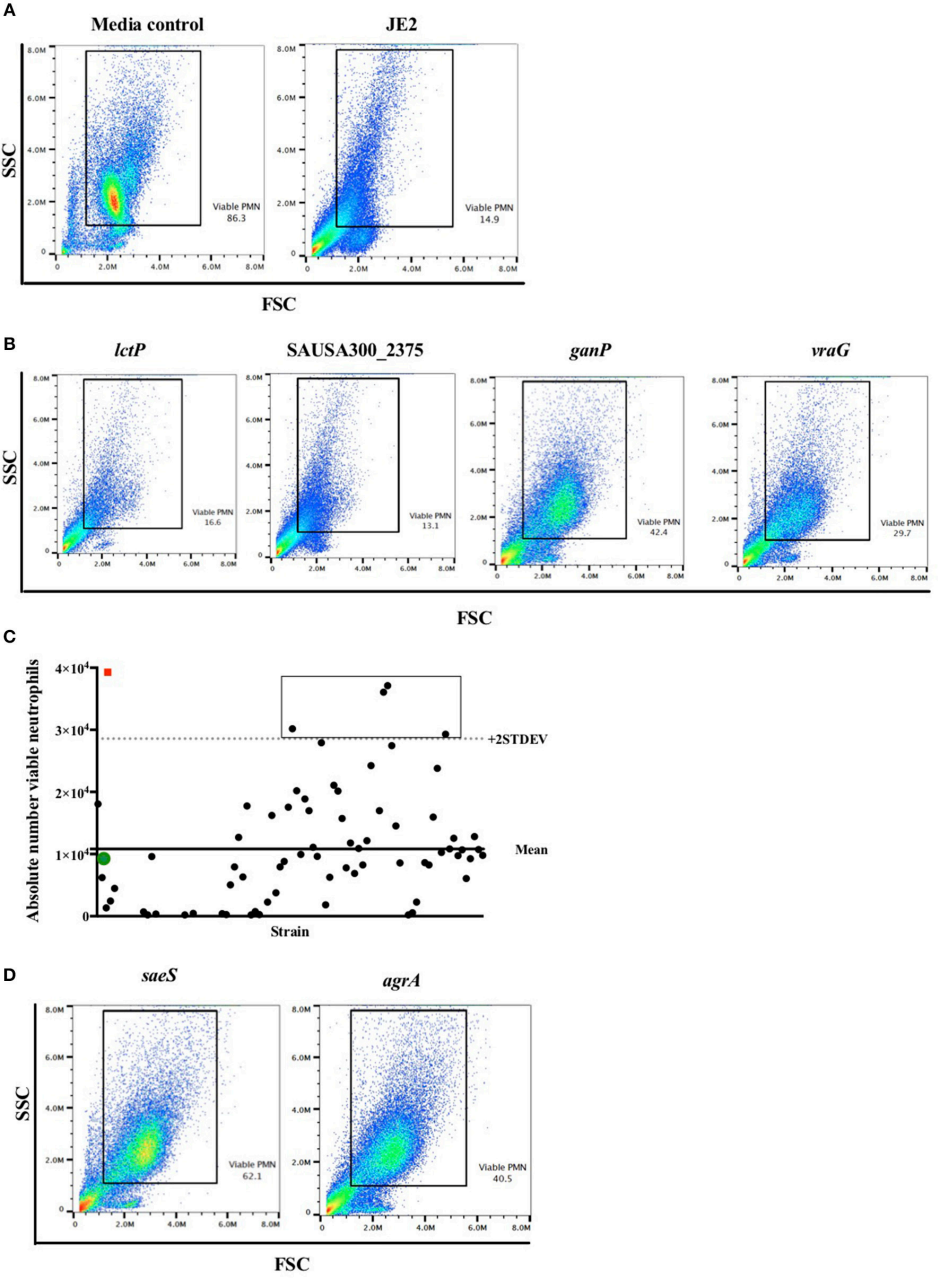
Flow cytometry viable neutrophil gating strategy identifies known lytic factors as proof of concept. Neutrophils were infected with individual NTML strains in 96-well plates at MOI 10 for 3 h following which they were subjected to flow cytometry. **(A)** Illustrative flow cytometry FSC/SSC dot plots for media control and JE2 infected samples. The rectangular gated region defines viable cells based on typical FSC and SSC profiles. Viable PMN (neutrophils) values are expressed as a percentage of events within the gated region. **(B)** Representative FSC/SSC dot plots of four randomly selected NTML strains for illustration. **(C)** Absolute number of viable neutrophils is calculated from viable gated region for each strain and plotted by strain (solid circles represent individual strains). Panel shows data from a single plate for illustration. Mean of all NTML strains (solid line) and mean +2 STDEV (dotted line) is shown. Open icons denote media treated (red square) and JE2-infected (green circle) controls for comparison. The rectangular box is drawn around strains identified as “hits.” **(D)** FSC/SSC dot plots of “hits” *saeS* and *agrA* mutant strains showing increased viable neutrophils compared to JE2 infected samples. Data is generated from a single representative experiment.

We took an unbiased approach to identifying novel genes related to the induction of neutrophil cell death by high-throughput and blinded screening of a genome-wide *S. aureus* mutant library. Neutrophils were infected with individual NTML strains in 96-well plates at MOI 10 for 3 h following which they were subjected to flow cytometry (Figure 1). A total of 1,920 strains were assayed across 20 plates (IDs: 1A-5D) over 11 non-consecutive days and 5 independent neutrophil donors (not pooled). Media treated and JE2 (WT) infected controls were included for each experiment. The absolute number of neutrophils was automatically calculated by an Attune flow cytometer for each strain. Figure 2 shows the gating strategy [based on known neutrophil FSC/SSC profiles (31)] used to determine the viable neutrophil population. Events outside the rectangular gate include cell debris and contaminating mononuclear and red blood cells. Viable neutrophils in media control treated conditions typically comprised between 80 and 90% of the total events (Figure 2A), which equated to absolute numbers of 28,944 ± 2,212 (mean ± SEM) across all experiments. A profound loss of viable neutrophils was seen in JE2 infected samples where absolute numbers equated to 14,936 ± 2,171 (mean ± SEM) across all experiments. Figure 2B shows dot plots of 4 representative NTML strains, showing 2 strains (L-lactate permease; *lctP* and ABC transporter ATP-binding/permease protein; SAUSA300_2375) that induced neutrophil cell death at comparable levels to JE2, and 2 “hit” strains (maltose ABC transporter, permease protein*, ganP*, and ABC transporter, permease protein*, vraG*) that were attenuated in their ability to induce neutrophil cell death. The attenuated strains have a greater number of events in the viable gated region compared to JE2. For each 96-well plate, the mean and 2 standard deviations from the mean (+2 STDEV) viable neutrophil number for all NTML strains were calculated (Figure 2C presents representative data from a single plate, 1A) and a box is drawn around strains identified as hits. A number of strains resulted in increased neutrophil lysis, where viable neutrophils were almost undetectable. While this is of great interest, and may reflect a loss of a negative regulatory mechanism of cell death, we did not further pursue these mutants, since our objective was to identify genes that played a positive role in the induction of neutrophil lysis. As a measure of screen robustness and “hit” sensitivity we were able to identify genes required for neutrophil lysis including the master regulators *saeS* and *agrA* (32, 33), both of which resulted in increased numbers of viable neutrophils (Figure 2D).

### The *S. aureus* Genes *purB, lspA*, and *clpP* Regulate Neutrophil Lysis

The objective of this screen was to identify genetic mutations that resulted in a defect in neutrophil cell death. Attenuated strains were identified as those with a viable neutrophil count >+2 STDEV of the plate mean and/or as those with a visibly different FSC/SSC profile (see Figure 2D). This latter strategy was adopted in order to maximize the number of hits identified in this initial screen round. Any false positives among these would be weeded out in the subsequent focused screen. 118 NTML strains were taken forward into a second round of focused screening, where each strain (incubated with neutrophils at MOI 10 for 3 h as per the primary screen) was tested in 3 independent experiments and presented as a box and whiskers plot (Supplemental Figure 1). As in the primary screen, attenuated hits were identified based on viable cell number >+2 STDEV of the plate mean. Thirty four strains were found to be attenuated (Supplemental Table 1). Strains were ranked based on the level of attenuation, that is to say the more viable neutrophils remaining, the greater the attenuation, and listed in descending order. As expected, some of the most attenuated strains included mutations in genes known to be profound modulators of neutrophil death, including leukocidins, *saeS*, and *agrA*. Note inclusion of the genes: *ganP* and *vraG* as highlighted in Figure 2B. To determine whether the attenuated neutrophil lysis was due to a bacterial growth defect and therefore reduced MOI, the number of viable neutrophils was correlated with OD_600_ following 3 h growth in BHI. There was no correlation between number of viable neutrophils and OD_600_ (Supplemental Figure 2), suggesting the extent of the attenuation was not because of differences in MOI.

**Figure 3:**
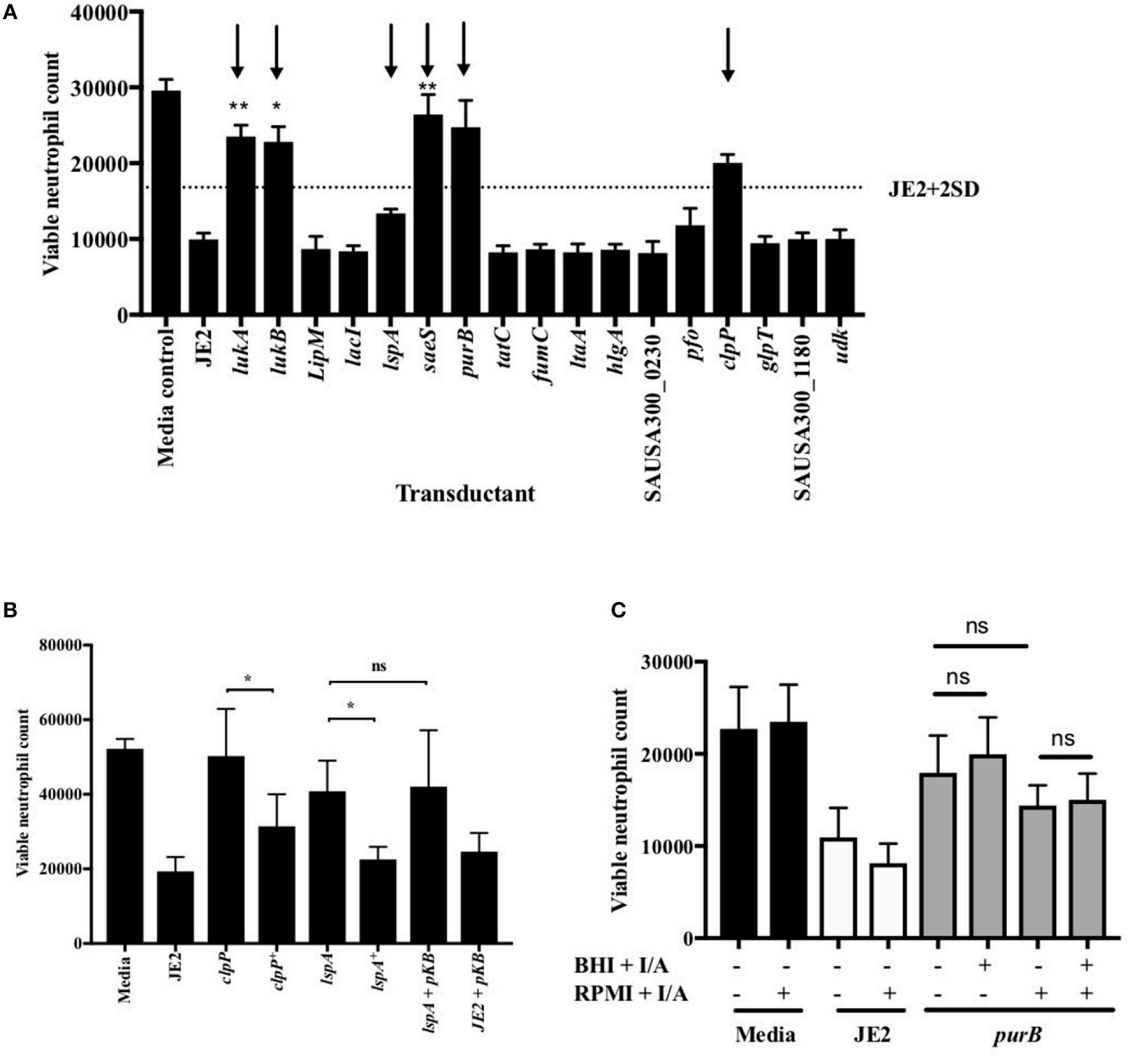
Validation of attenuation by genetic transduction and complementation. **(A)** Neutrophils were co-incubated with media, JE2 or *S. aureus* transductants at MOI 10 for 3 h, followed by flow cytometry. Viable neutrophil counts were generated as previously described. JE2 + 2D is illustrated by dotted line and attenuated strains to be taken forward indicated by arrows (*n* = 3). **(B)** Neutrophils were co-incubated with media, JE2, transductants, or complements (indicated by ^+^) at MOI 10 for 3 h. JE2 and *lspA* transductant was also incubated in the presence empty pKasbar plasmid (pKB) (*n* = 6). **(C)** Chemical complementation of *purB* mutant was performed by addition of both inosine and adenine (I/A) to solid BHI agar during bacterial growth or RPMI media during neutrophil infection (indicated by single ^+^) or both (indicated by double ^++^) at a final concentration of 0.02 mg/ml. The absence of I/A in RPMI or BHI is indicated by (^−^). Neutrophils were incubated with *S. aureus* for 3 h at MOI 10 and viable neutrophils enumerated (*n* = 3). Data expressed at mean ± SEM and analyzed by ANOVA with Bonferoni post-test ^*^p < 0.05, ^**^p < 0.01. Comparisons were between JE2 and transductant **(A)** or as indicated **(B,C)**.

**Figure 4:**
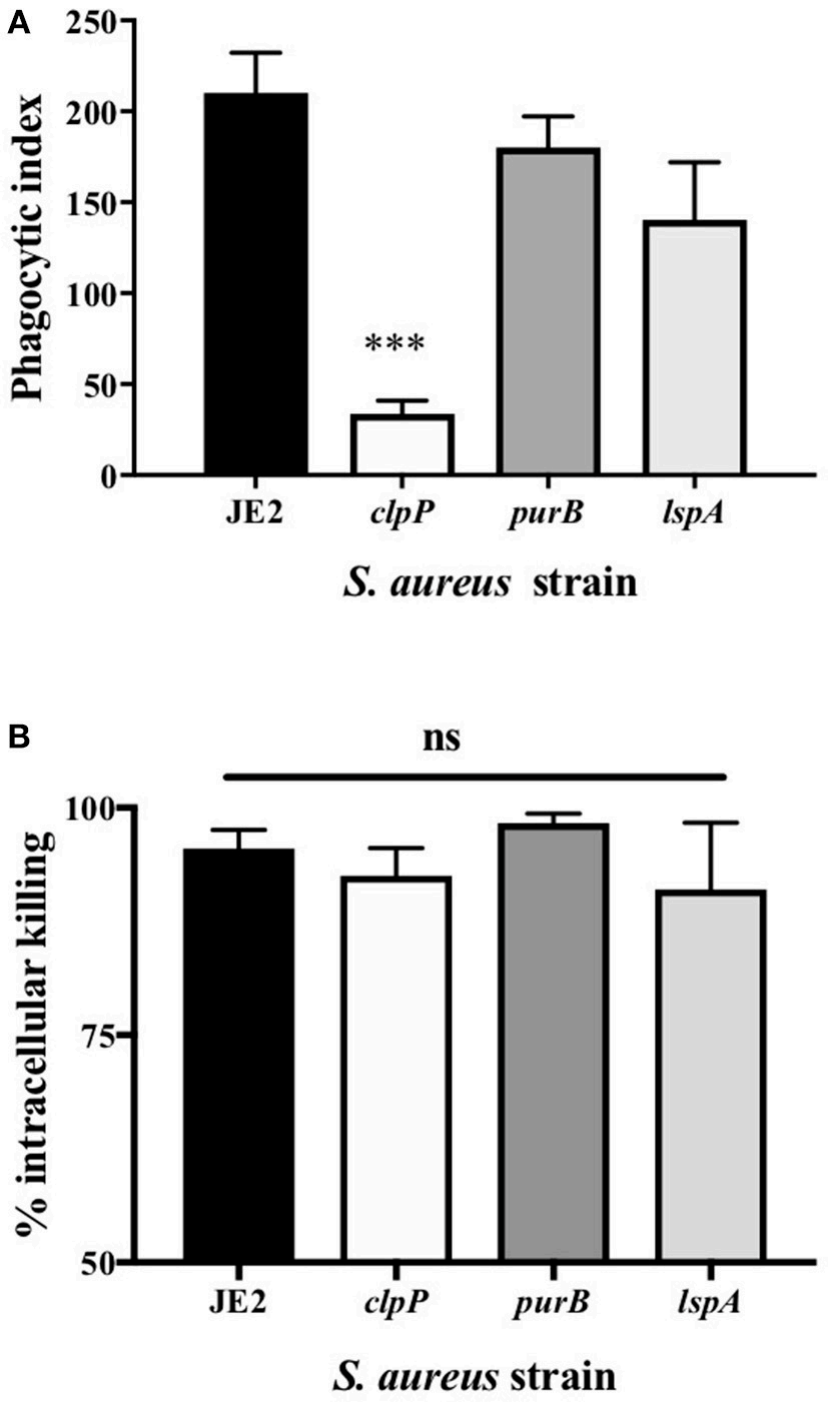
Loss of *clpP* leads to a reduction in phagocytosis but there is no impact on killing by neutrophils **(A)** Neutrophils were incubated with JE2 and *clpP, purB*, or *lspA* strains at MOI 10 for 1 h. Cytocentrifuge slides were prepared and the phagocytic index assessed by light microscopy. Data are expressed as mean ± SEM (*n* = 6). **(B)** Neutrophils were incubated with JE2, *ClpP, purB*, or *lspA* strains at MOI 5 and an intracellular killing assay performed. Graph shows % killing of *S. aureus* by calculating the reduction in the number of viable bacteria at 120 min compared to 30 min for each strain. Data expressed as mean ± SEM (*n* = 3). Data were analyzed by ANOVA with Bonferoni post-test ^***^p < 0.001.

Of the attenuated strains identified in the second screen, 17 of the most attenuated mutants were further validated in transduction studies. To do this the transposon insert for each strain identified in the screen was transduced back into the parent strain (*S. aureus* JE2) and transductants were rescreened to establish that the mutant phenotype was specifically associated with each Tn insertion. Neutrophils were infected with transductants at an MOI 10 for 3 h and viable neutrophils enumerated by flow cytometry as above. Transductants (3 clones of each mutation) of 5 original mutants were attenuated (>+2STDEV of the WT strain, JE2) including the known cytolytics *lukAB* and *saeS*, as well as adenylosuccinate lyase (*purB*) and the ATP-dependent Clp protease proteolytic subunit (*ClpP*, Figure 3A). *ClpP* in addition to the lipopeptidase *lsp*A, which was moderately attenuated, were taken further into genetic complementation studies. Genetic complements of *lspA* and *clpP* (indicated by ^+^) were able to induce neutrophil cell lysis to levels comparable to JE2 (Figure 3B, ^*^p < 0.05 mutant vs. complement). The integration of empty pKB had no effect on neutrophil lysis. Representative flow cytometry plots showing FSC/SSC profiles and viable neutrophils are shown in Supplemental Figures 3A–D. To determine neutrophil lysis by an alternative method, we performed an LDH assay and show that *clpP* and *purB*, but not *lspA* resulted in significantly attenuated cytotoxicity (Supplemental Figure 3E). The lack of attenuation by *lspA* in this assay may reflect membrane damage leading to leakage of LDH, but without complete cell lysis (as indicated by flow cytometry plots that are consistent with intact cells, Supplemental Figure 3B). Since PurB is required for purine synthesis, the *purB* mutant was chemically complemented by the addition of adenine and inosine to BHI agar during overnight growth of *S. aureus* and/or during neutrophil infection. The presence of adenine and inosine to BHI had no effect on neutrophil viability. Addition of adenine and inosine to RPMI during the infection partially restored the ability of the *purB* mutant to induce neutrophil cell lysis, although this was not statistically significant (Figure 3C). This indicates that *S. aureus*-induced neutrophil cell death is dependent on purines and highlights the specific requirement of purines during the infection period. Phagocytosis assays revealed the *clpP* mutant was phagocytosed significantly less avidly than the *purB* and *lspA* mutants (Figure 4A), but all strains were killed equally well by human neutrophils (Figure 4B). This suggests that the attenuation in neutrophil cell death is not a result of altered killing of *S. aureus*.

### PurB and ClpP Are Necessary for Bacterial Replication and Pathogenesis in a Zebrafish Embryo Infection Model

**Figure 5:**
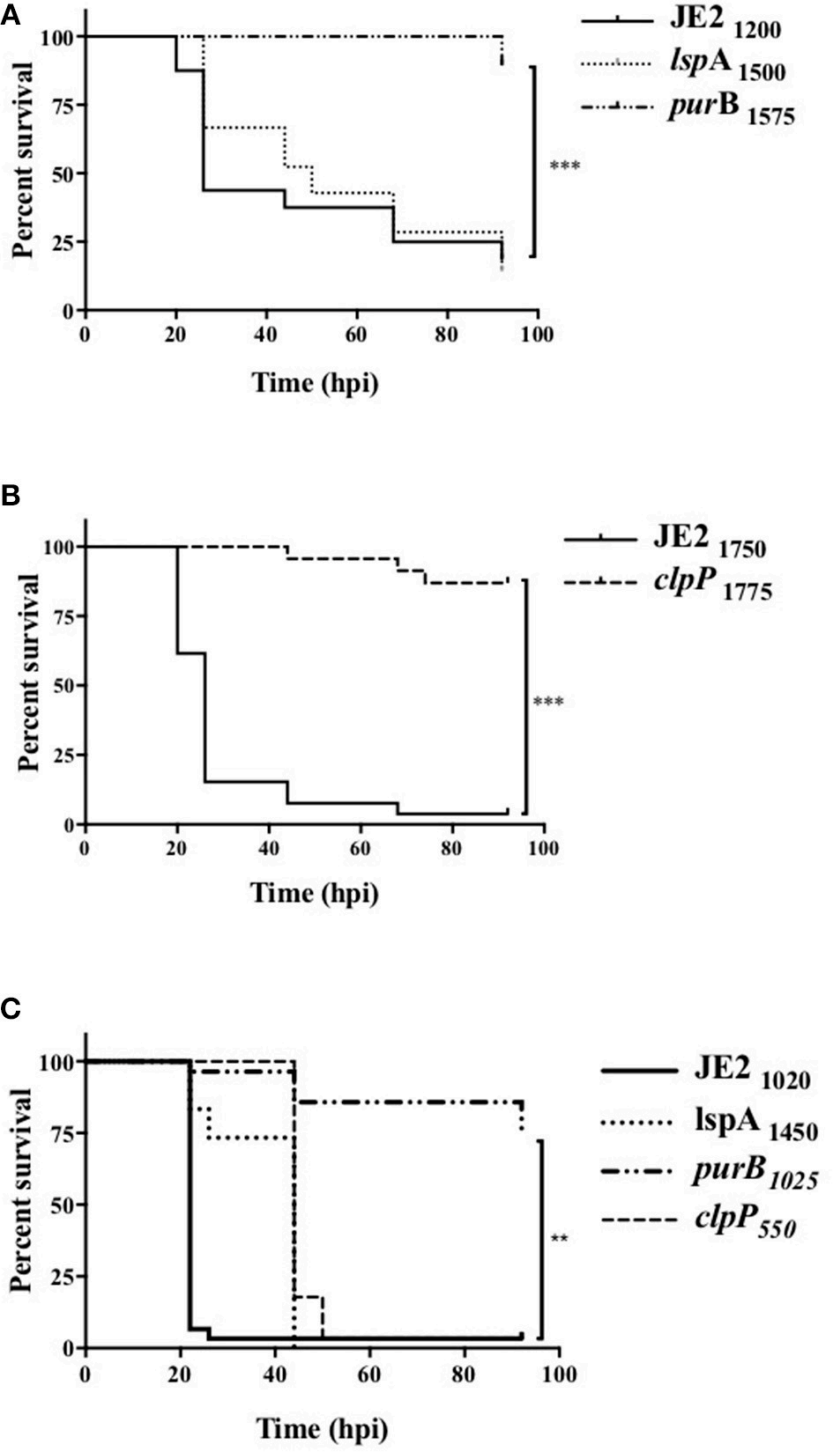
Altered pathogenicity of mutants in zebrafish models of *in vivo* infection. Zebrafish embryos at 24 hpf were injected into the circulation valley with JE2, *lspA, purB*, or *clpP* mutants **(A,B)** transductants. Exact inoculum was determined retrospectively by Miles Misra assays and indicated as subscript values. **(C)** At 30 min post-fertilization zebrafish embryos were injected with a *pu.1* morpholino to delete phagocytes. Embryos were injected at 32 hpf into the circulation valley with the indicated inoculum of JE2*, lspA, purB*, or *clpP* strains. Survival curves over 92 h post-infection (hpi) were calculated by Kaplan-Meier analysis, statistical significance is indicated by ^**^p < 0.01, ^***^p < 0.001.

Since *clpP, lspA*, and *purB* mutants were defective in causing neutrophil lysis and therefore may not overcome neutrophil defenses during infection, we hypothesized these strains would have altered pathogenicity *in vivo*. To test this, mutants were studied in a zebrafish embryo infection model (29). Survival rate in PBS injected embryos was >90% (data not shown). As expected, infection with JE2 resulted in profound embryo death (Figures 5A,B, solid line). The *lspA* mutant (dotted line) caused significant mortality at rates comparable to JE2 (Figure 5A). In contrast, *purB* and *clpP* mutants (dashed lines) failed to kill embryos, with almost maximum survival at 92 h (Figures 5A,B). To determine whether a phagocyte response was critical for host immunity to strains, myeloid cells were depleted in zebrafish embryos with a morpholino to pu.1 (30, 34). Consistent with previous studies (34), depletion of neutrophils increased the speed at which embryos died following infection with JE2 with >95% embryos dead by 24 h (Figure 5C). Compared to JE2, all mutants delayed embryo death by 24 h but with the exception of *purB*, all went on to completely overcome the zebrafish by 48 h. The *clpP* mutant was able to mount an overwhelming infection in the absence of neutrophils but not in the presence of neutrophils which suggests neutrophils are key in the control of this strain.

**Figure 6:**
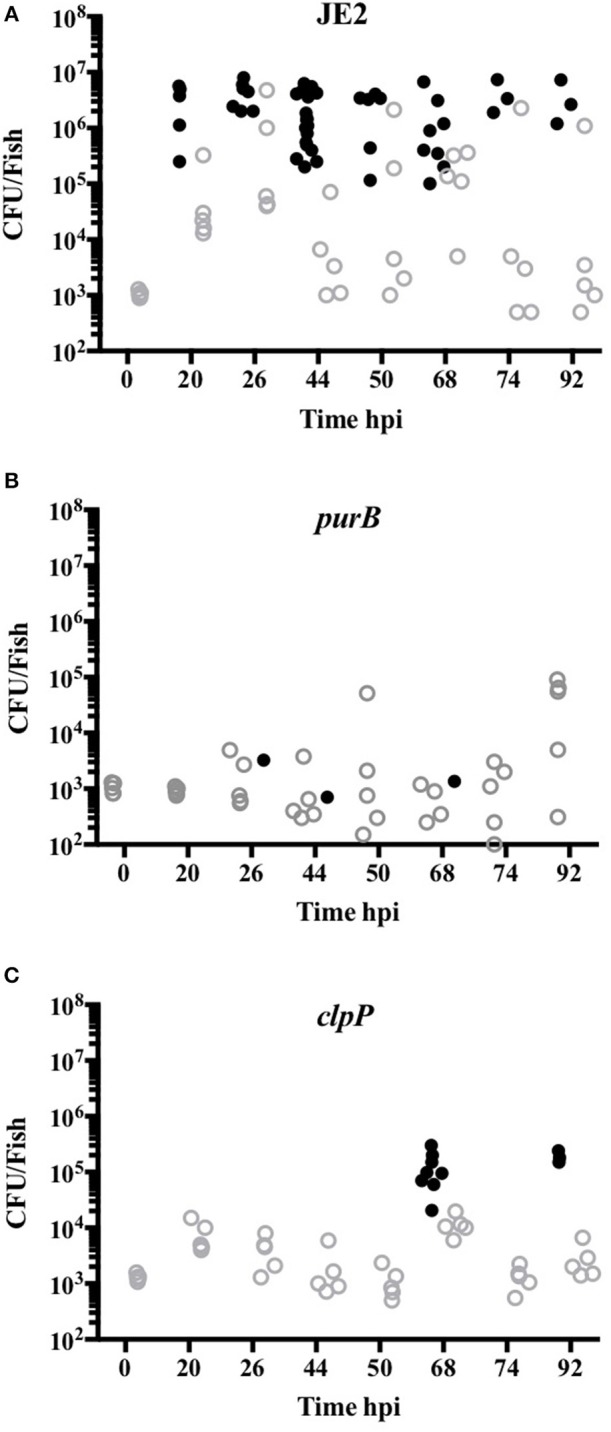
*purB* and *clpP* mutants fail to replicate in zebrafish models of *in vivo* infection. Zebrafish embryos at 24 hpf were injected into the circulation valley with JE2 **(A)**, *purB* **(B)** or *clpP* **(C)** at 950, 1025 and 1275 CFU, respectively. CFU counts from up to five viable (open gray circles) and non-viable (closed black circles) homogenized embryos at selected time points were determined by Miles Misra.

To define whether increased zebrafish survival was associated with lack of bacterial replication, CFU counts from viable and dead embryos were determined. CFU counts of up to 10^7^ were recovered from dead embryos infected with JE2, even at early timepoints, indicating rapid replication *in vivo* (Figure 6A). For mutants that were less efficient at killing zebrafish (*clpP* and *purB*), CFU counts in both dead and viable zebrafish were markedly lower at between 10^2^ and 3 × 10^5^ (Figures 6B,C). The results suggest that *clpP* and *purB* mutants had limited capacity to replicate within embryos, and were therefore unable to overcome the zebrafish. As seen in other zebrafish bloodstream infection models, none of the *S. aureus* strains were completely cleared from any viable embryo over the timecourse studied (29).

## Discussion

The importance of neutrophils both in the control of colonization and during active *S. aureus* infection makes them an ideal target for bacterial immune evasion strategies (5, 6). Preserving neutrophil function during infection by preventing *S. aureus*-induced cell death is therefore an attractive therapeutic strategy and here we describe three genes (*purB, clpP*, and *lspA*) with previously unidentified roles in neutrophil cell death. Mutations in two of these genes, *purB* and *clpP*, led to significantly reduced pathogenesis in a zebrafish model of infection. Virulence for *clpP* but not *purB* was restored by depleting neutrophils in zebrafish. Our work suggests that ClpP is a key element of *S. aureus* pathogenicity and therapeutically targeting ClpP may improve outcome during *S. aureus* infections (35–37).

*Staphylococcus aureus* is well-known to cause a lytic or necrotic like neutrophil cell death via factors that directly damage the cell (38–41). It is unlikely however that any of the gene products identified in our study are directly lytic to neutrophils. In the case of PurB, a deficiency in purine biosynthesis and therefore a failure to replicate and express virulence factors in a nutrient poor environment is likely to explain the reduction in neutrophil lysis. This was in part confirmed by the addition of adenine and inosine during infection of neutrophils, which partially increased neutrophil lysis. *S. aureus purB* mutants have been recently shown by our group to have reduced pathogenesis, and *purA* mutants were found to be attenuated in a *S. aureus* murine abscess study (16, 42). In support of this we observed an attenuated phenotype of the *purA* mutant in the primary screen and this was taken forward into the secondary screen. The level attenuation however, was not as robust as *purB* and therefore was not carried forward into further studies. < underline >We found the attenuated virulence of the *purB* mutant in zebrafish was unaffected by the absence of neutrophils. This further suggests that a fundamental deficiency to replicate, rather than a failure to overcome innate immunity, was the cause of the attenuation.

The lipoprotein signal peptidase product of *lspA* is localized in the bacterial membrane and required for biogenesis of bacterial lipoproteins (43). Lipoproteins themselves can play essential roles in host-pathogen interactions, for example as pathogen associated molecule patterns (PAMPs) acting via TLRs (44–46). Bacterial lipoproteins can induce apoptosis in other cell types (47). Although we measure a non-apoptotic cell death in this study, it is conceivable that activation of an apoptosis programme by lipoproteins in concert with other death inducing toxins may lead to an overall net effect of lysis and therefore by removing lipoproteins a lytic pathway is prevented. Failure to mature lipoproteins due to loss of LspA or other peptidases has previously been shown to impair growth and pathogenicity, but our study is the first to describe a specific immune modulating role for LspA in *S. aureus* (44, 48). In murine models of *S. aureus* infection an *lsp* mutant failed to induce disease (44) but we show no attenuation in zebrafish embryos. Since *S. aureus* has evolved as a human associated organism it is possible that this tropism may extend to the targets of LspA, explaining the species specificity of the observed effect.

The principal function of Clp proteases is protein degradation, although they are associated with a number of physiological processes (36). *ClpP* mutants are highly susceptible to stress and the attenuated phenotype caused by the *clpP* mutant in our study may be due to lack of stress adaptation in the neutrophil phagosomal environment. *S. aureus* is adept at escape from the richly anti-microbicidal phagosome (49, 50). ClpP deficient *Legionella pneumophila* fails to escape from the endosome-lysosomal pathway in a macrophage cell line and *S. aureus* mutants are unable to replicate intracellularly (35, 51). Interestingly this has also been shown for *lspA* in *Listeria monocytogenes* (52). It is possible that containment within the neutrophil phagosome may prevent post-phagosomal cell lysis for these mutants, but further studies are required. The *clpP* mutant was not phagocytosed as readily, which may be as a result of an impairment in lipoprotein-dependent recognition by neutrophils, leading to a reduction in post-phagocytic cell lysis.

ClpP regulates the expression of a number of bacterial virulence factors such as hemolysin, which may in part account for the attenuation in neutrophil cell death seen in our study (24, 53). As a result, *clpP* mutants are found to be less virulent in animal models (53, 54). We also show attenuation *in vivo*, where the *clpP* mutant fails to replicate in zebrafish embryos, and which is supported by others that demonstrate a failure to replicate in the host (35). A growing number of studies highlight the therapeutic potential of targeting ClpP. A recent study describes a selective, small-molecule inhibitor of ClpP, identified via high-throughput screening and which attenuates virulence in mouse models of *S. aureus* USA300 infection (55). Our work and others suggest therapeutically targeting ClpP may have great promise in treating invasive *S. aureus* infections. In conclusion, our work identifies genetic components underpinning *S. aureus* pathogenesis and provides further evidence for the complex interaction between pathogen and host. These findings provide a greater insight into how this commensal organism breaches innate immune barriers during infection.

## Author Contributions

DY, SF, and LP wrote the manuscript. DY, YH, IJ, and LC performed the experiments. All authors contributed to experimental design and data analysis.

### Conflict of Interest Statement

The authors declare that the research was conducted in the absence of any commercial or financial relationships that could be construed as a potential conflict of interest.
