# Supplementary material for: A Genome-Wide Screen Identifies Factors Involved in S. aureus-Induced Human Neutrophil Cell Death and Pathogenesis
Source: Front Immunol. 2019 Jan 31;10:45. doi: 10.3389/fimmu.2019.00045 (PMC6365652; doi:10.3389/fimmu.2019.00045)
Supplement: Supplementary file 1 [file Table_1.docx]

**Supplemental Table 1. NTML strains identified as attenuated in second round of screening.** The level of attenuation was calculated from arbitrary scores based on a reduction in viable neutrophils number (score of 1) or visible difference in FSC/SSC profiles (score of 1) or both (score of 2.5). A score of 7.5 (2.5 from each of 2 experiments) was the maximum value attributable. Genes are ranked in descending order of attenuation.

| **Protein name** | **Accession number** | **Gene name** | **Level of attenuation** |
| --- | --- | --- | --- |
| Leukocidin/Hemolysin toxin family protein | SAUSA300_1974 | *lukA* | 7.5 |
| Aerolysin/leukocidin family protein | SAUSA300_1975 | *lukB* | 7.5 |
| Octanoyltransferase LipM | SAUSA300_1494 | *lipM* | 7.5 |
| LacI family transcriptional repressor | SAUSA300_0265 | *lacI* | 6 |
| lipoprotein signal peptidase | SAUSA300_1089 | *lspA* | 6 |
| pyruvate dehydrogenase E1 component, beta subunit | SAUSA300_0994 | *pdhB* | 6 |
| sensor histidine kinase SaeS | SAUSA300_0690 | *saeS* | 5 |
| adenylosuccinate lyase | SAUSA300_1889 | *purB* | 5 |
| Sec-independent protein translocase TatC | SAUSA300_0347 | *tatC* | 5 |
| fumarate hydratase, class II | SAUSA300_1801 | *fumC* | 5 |
| Probable glycolipid permease LtaA | SAUSA300_0917 | *ltaA* | 5 |
| gamma-hemolysin component A | SAUSA300_2365 | *hlgA* | 5 |
| putative membrane protein | SAUSA300_0230 |  | 4.5 |
| uracil permease | SAUSA300_1092 | *pyrP* | 4.5 |
| accessory gene regulator protein A | SAUSA300_1992 | *agrA* | 3.5 |
| pyruvate ferredoxin oxidoreductase, alpha subunit | SAUSA300_1182 | *pfo* | 3.5 |
| ATP-dependent Clp protease proteolytic subunit | SAUSA300_0752 | *clpP* | 3.5 |
| glycerol-3-phosphate transporter | SAUSA300_0337 | *glpT* | 3 |
| Conserved hypothetical protein | SAUSA300_1180 |  | 3 |
| uridine kinase | SAUSA300_1568 | *udk* | 3 |
| amino acid permease | SAUSA300_0566 |  | 2.5 |
| Phosphoesterase | SAUSA300_1051 |  | 2.5 |
| S-ribosylhomocysteinase | SAUSA300_2088 | *luxS* | 2.5 |
| 3-dehydroquinate synthase | SAUSA300_1356 | *aroB* | 2.5 |
| phiSLT ORF2067-like protein, phage tail tape measure protein | SAUSA300_1393 |  | 2 |
| maltose ABC transporter, permease protein | SAUSA300_0210 | *ganP* | 2 |
| ABC transporter, permease protein | SAUSA300_0648 | *vraG* | 2 |
| acetyltransferase, GNAT family | SAUSA300_0662 |  | 2 |
| putative transposase | SAUSA300_1732 |  | 2 |
| hypothetical protein | SAUSA300_2042 |  | 2 |
| hypothetical protein | SAUSA300_0957 |  | 2 |
| D-isomer specific 2-hydroxyacid dehydrogenase family protein | SAUSA300_1843 |  | 2 |
| glutamine amidotransferase subunit PdxT | SAUSA300_0505 | *pdxT* | 2 |
| phosphopentomutase | SAUSA300_0141 | *deoB* | 2 |
