# Supplementary figures and images for: A Genome-Wide Screen Identifies Factors Involved in S. aureus-Induced Human Neutrophil Cell Death and Pathogenesis
Source: Front Immunol. 2019 Jan 31;10:45. doi: 10.3389/fimmu.2019.00045 (PMC6365652; doi:10.3389/fimmu.2019.00045)

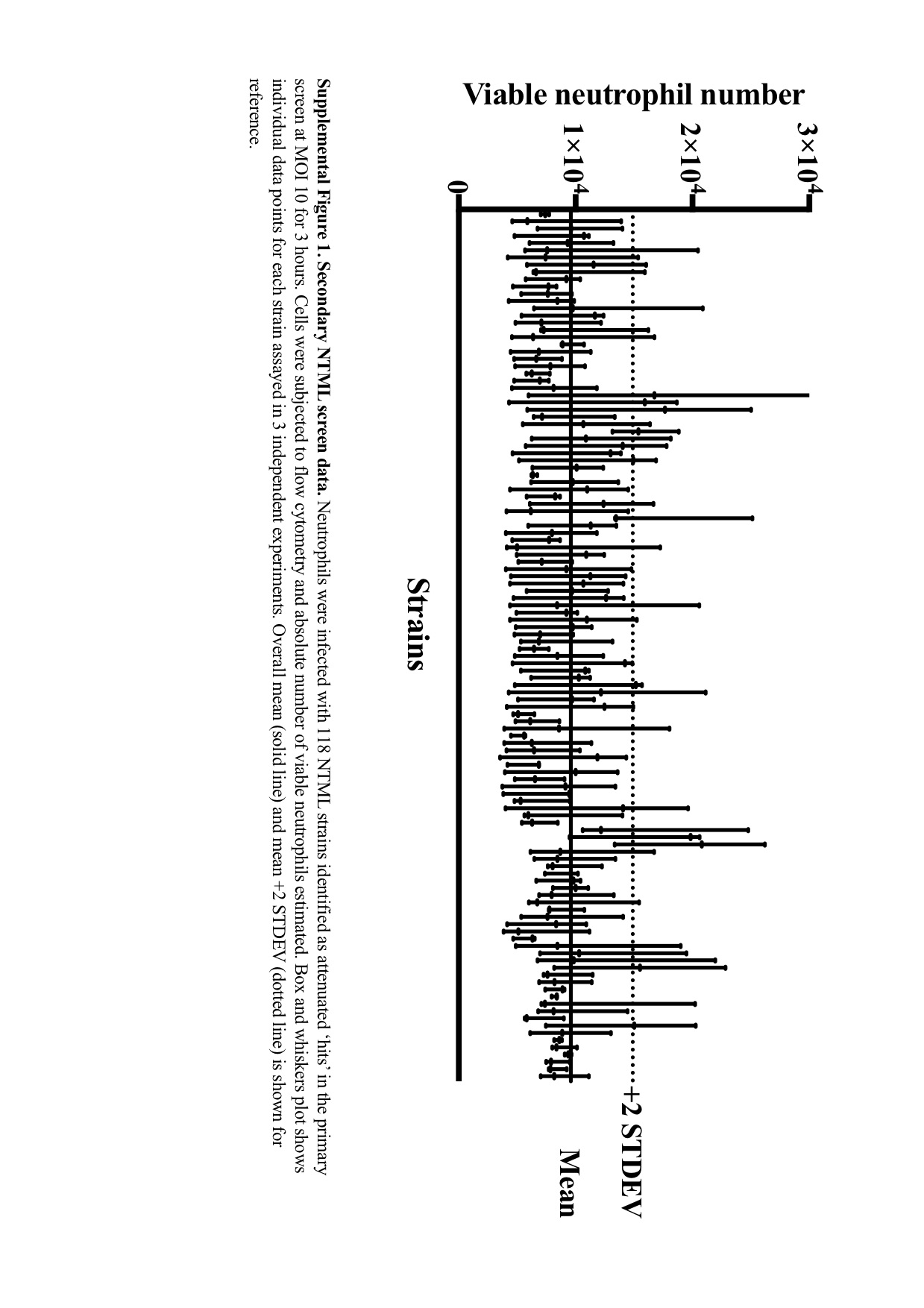

Supplement: Supplementary file 2 [file Image_1.jpeg]

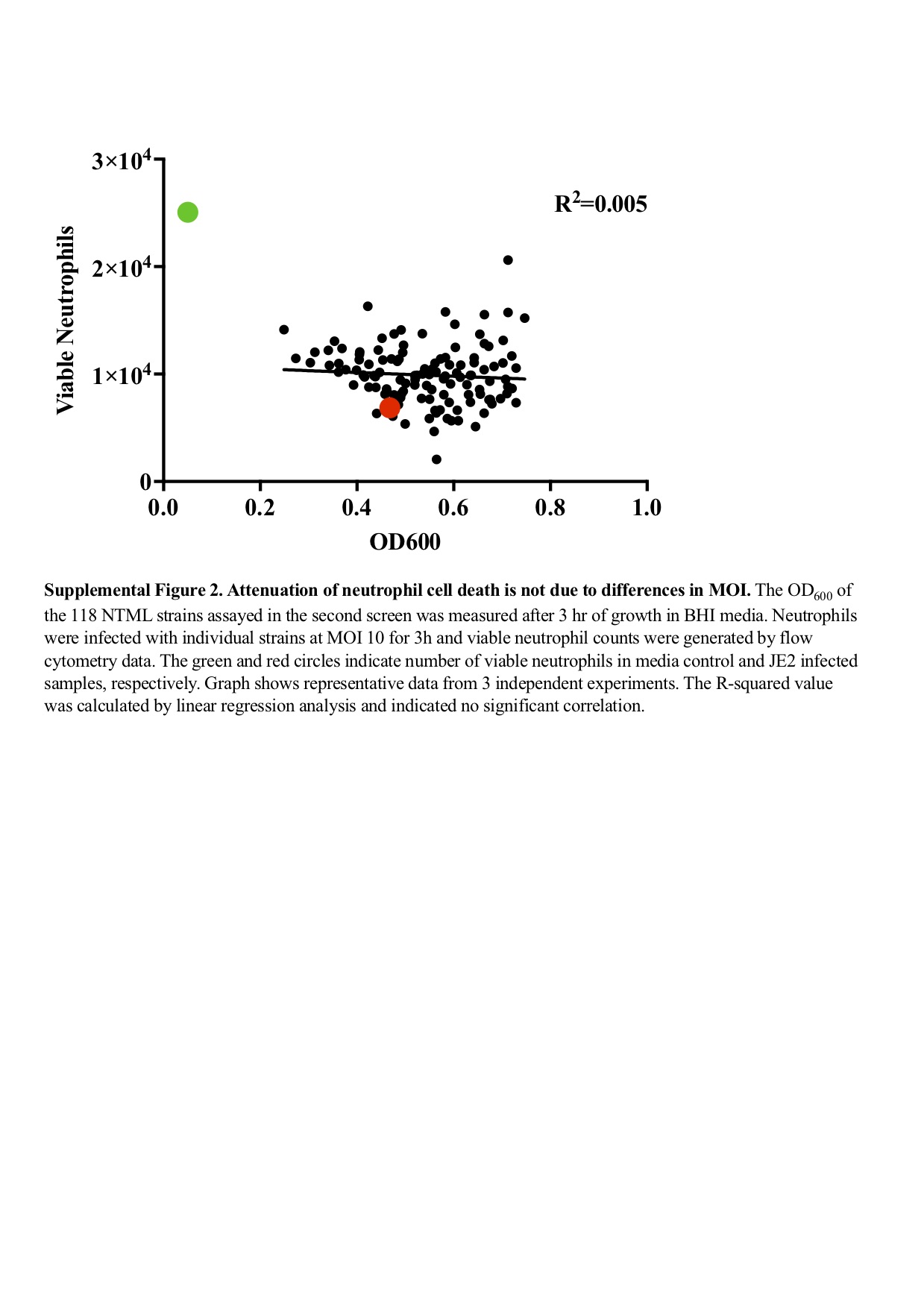

Supplement: Supplementary file 3 [file Image_2.JPEG]

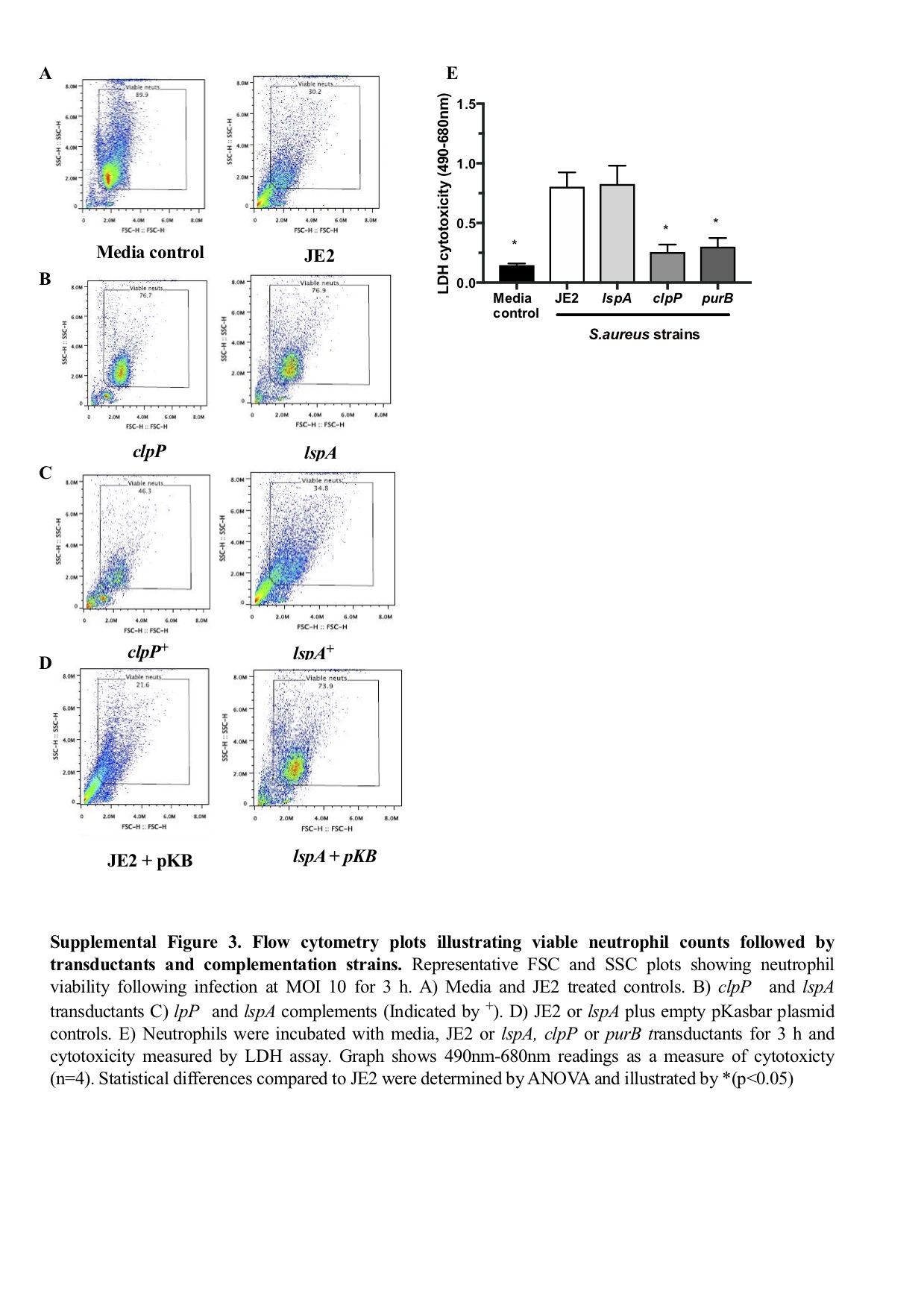

Supplement: Supplementary file 4 [file Image_3.JPEG]
